# Do grasses have meristemoids?

**DOI:** 10.1111/nph.70389

**Published:** 2025-07-14

**Authors:** Laura Serna

**Affiliations:** ^1^ Department of Environmental Sciences Institute of Environmental Sciences (ICAM), Earth and Space Science (ESS) Research Group, University of Castilla‐La Mancha 45071 Toledo Spain

**Keywords:** Arabidopsis, FAMA, grasses, guard mother cell, meristemoid, MUTE, stem cells

## Disclaimer

The New Phytologist Foundation remains neutral with regard to jurisdictional claims in maps and in any institutional affiliations.

Self‐renewal is the process by which stem cells divide to produce at least one daughter cell with the same capacity for self‐renewal and differentiation as the parent cell (He *et al*., 2009; Greb & Lohmann, 2016). This daughter cell eventually differentiates to build up new tissues and/or cells. Stem cells exist in plants and animals, sharing, despite their substantial divergence at the molecular level, surprising similarities (Sablowski, 2004; Heidstra & Sabatini, 2014). Their enormous potential to produce new cells to grow or replace specialized tissues is having a strong impact on regenerative medicine (Hoang *et al*., 2022) and in agriculture for crop improvement (Lindsay *et al*., 2024). Stem cells are maintained in specialized microenvironments, which are known as stem cell niches (Sablowski, 2004; Mitsiadis *et al*., 2007; Heidstra & Sabatini, 2014). In plants, these niches are mainly located within the meristems and they produce most postembryonic development (Sablowski, 2004; Aichinger *et al*., 2012; Heidstra & Sabatini, 2014; Hong & Fletcher, 2023). Plant stem cells are also dispersed in the epidermis of the developing leaf and cotyledon of many plant species. These stem cells, named meristemoids (Ms), are responsible for triggering stomatal development after they cross a critical cell size threshold (Gong *et al*., 2023).

**Fig. 1:**
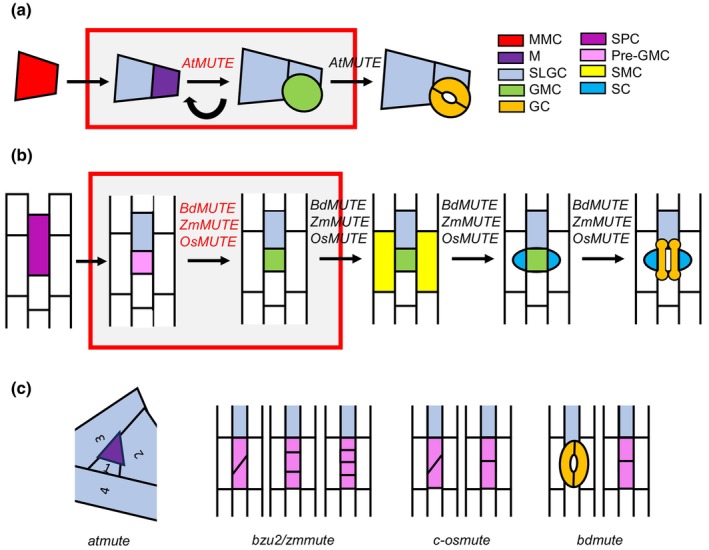
*AtMUTE* and grass *MUTE* orthologs promote a pre‐GMC stage in Arabidopsis and grasses. (a) In Arabidopsis, a MMC cell divides asymmetrically to produce a M and a larger SLGC. Meristemoids are self‐renewing cells, in which one of the daughter cells retains the properties of the parent cell undergoing asymmetric divisions until it assumes GMC identity. The GMC then divides symmetrically to produce the two kidney‐shaped GCs. *AtMUTE* promotes the transition from the M to the GMC. Therefore, the timing of *AtMUTE* expression determines the number of amplifying divisions that Ms undergo. *AtMUTE* also triggers paired GCs formation from the GMC. (b) Stomatal development in grasses starts with an asymmetric division from a SPC, which produces a pre‐GMC and a SLGC. The pre‐GMC assumes GMC identity. Before GMC division, cells from files on either side of the GMC are fated to become SMCs. SMCs then divide asymmetrically to produce SCs that make direct contact with the GMC. When the GMC is flanked by the two SCs, they symmetrically divide giving rise to the two dumbbell‐shaped GCs. Early expression of grass MUTE genes, among other functions, likely represses the stem cell character of the pre‐GMC, promoting its transition to GMC fate without the need to undergo amplifying divisions. (c) Drawings of stomatal phenotypes of *atmute*, *bzu2/zmmute*, *c‐osmute* and *sid/bdmute*, showing that *AtMUTE* and grass *MUTE* genes repress stem cell character of the SPCs. GC, guard cell; GMC, guard mother cell; M, meristemoid; MMC, meristemoid mother cell; SC, subsidiary cell; SLGC, stomatal lineage ground cell; SMC, subsidiary mother cell; SPC, stomatal precursor cell.

Meristemoids, therefore, are self‐renewing cells, in which one of the daughter cells can exhibit stem properties. In the model plant Arabidopsis, they arise in a basipetal direction from asymmetric cell divisions (entry divisions) of protodermal cells called meristemoid mother cells (MMCs; Serna & Fenoll, 2000; Bergmann & Sack, 2007; Peterson *et al*., 2010; Vaten & Bergmann, 2012; Fig. 1a). These Ms, which are the smaller daughter cells, can either directly assume guard mother cell (GMC) fate or undergo several asymmetric divisions in an inward spiral (amplifying divisions), yielding a larger cell and a smaller M that maintains its self‐renewal activity. Finally, the M assumes GMC identity. The GMC undergoes an equal and symmetric cell division that generates the two kidney‐shaped guard cells (GCs). The larger cells that result from the asymmetric divisions of MMCs or Ms, named larger stomatal lineage ground cells (SLGC), can directly differentiate into pavement cells or execute a division (spacing division) to establish an additional M. This developmental process is responsible for generating almost all the epidermal cells in the Arabidopsis leaves (Geisler *et al*., 2000).

In grasses, among them the model Brachypodium (*Brachypodium distachyon*) and the cereal crops maize (*Zea mays*) and rice (*Oryza sativa*), the stomatal precursor cells (SPCs) form from the base to the tip of stomatal‐forming cell files, and they undergo a single transverse division (entry division) producing a distal cell that gives rise, without undergoing amplifying divisions and after a symmetric division, to two dumbbell‐shaped GCs (Stebbins & Shah, 1960; Serna, 2011; Hepworth *et al*., 2018; Nunes *et al*., 2020; Fig. 1b). Some authors have called this immediate precursor of the stoma GMC, which has led to the speculation that grasses do not form Ms (Peterson *et al*., 2010; Hepworth *et al*., 2018; Serna, 2020). This stomatal precursor divides to produce the two GCs only after cells from ontogenetically unrelated files acquire subsidiary mother cell (SMC) identity and undergo a longitudinal division producing the lateral subsidiary cells (SCs). Like SMCs, the stomatal precursor divides with its cell division plane being parallel to the main axis of the leaf. The proximal cell arising from the entry division (SLGC) could be a way to adjust the stomatal pattern, spacing stomata within a row and perhaps achieving an adequate distance with stomata located in neighboring files.

In addition to regulating the transition from GMC to GCs (Han *et al*., 2018), one of the key factors that represses the stem cell character of the Ms in Arabidopsis is the basic helix–loop–helix (bHLH) transcriptional factor AtMUTE (MacAlister *et al*., 2007; Pillitteri *et al*., 2007). *AtMUTE* expression and the localization of the protein encoded by this gene are restricted, in addition to the GMC, to a subset of Ms (MacAlister *et al*., 2007; Pillitteri *et al*., 2007). These Ms will presumably undergo GMC transition (Serna, 2013). In these Ms, *AtMUTE* integrates with cell cycle machinery upregulating the cyclin‐dependent kinase inhibitor SIAMESE‐RELATED4 (SMR4), which represses stem cell divisions by inhibiting the G1‐specific D‐Type Cyclin CYCD3;2 (Han *et al*., 2022). SMR4 also permits MUTE‐induced G1 cyclin CYCD5;1 to proceed to a terminal GMC division. In grasses, the primary role of MUTE proteins, which laterally move from the immediate SPCs where their genes are expressed to the neighboring epidermal cell files, is promoting SC recruitment (Raissig *et al*., 2017; Wang *et al*., 2019). Grass *MUTE* genes, in addition to regulating other steps of stomatal development (Serna, 2025), also inhibit cell divisions of the pre‐GMCs and perhaps the expression of a possible stem cell character of these cells. The integration of grass *MUTE* genes with the cellular machinery is not known. Here, I focus on what underlies the absence of manifestation of a SPC self‐renewal stage in grasses.

## *AtMUTE* represses the stem cell character of Ms and promotes GMC fate

In Arabidopsis, stomata develop from Ms, which usually after a few divisions transition to the GMC fate. Studies of cell divisions through time using serial imprints, clonal analysis and long‐term confocal time‐lapse imaging revealed that the number of self‐renewal divisions that undergo the Ms before producing the paired GCs is not fixed but varies from zero to three within a given ecotype and plant organ (Berger & Altmann, 2000; Geisler *et al*., 2000; Serna *et al*., 2002; Robinson *et al*., 2011; Serna, 2013).

But what are the molecular bases of this plasticity? Why do some Ms divide, while others are immediately fated to GMCs? In the absence of *AtMUTE* function, stomata are replaced by Ms, which arrest after having undergone an excess of self‐renewing cell divisions that occur in an inward‐spiral pattern (Fig. 1c; MacAlister *et al*., 2007; Pillitteri *et al*., 2007). In addition, *AtMUTE* overexpression converts all epidermal cells into stomata (MacAlister *et al*., 2007; Pillitteri *et al*., 2007). This is telling us that *AtMUTE* terminates the self‐renewing state of the Ms and promotes GMC fate (MacAlister *et al*., 2007; Pillitteri *et al*., 2007). This gene also positively regulates the transition from GMC to GCs (Han *et al*., 2018). Its expression and the localization of the protein encoded by *AtMUTE* are restricted to a subset of Ms and to the GMCs (MacAlister *et al*., 2007; Pillitteri *et al*., 2007). These Ms presumably will not divide, and they will undergo GMC transition (Serna, 2013). Thus, it is the variability in the timing of *AtMUTE* expression during the development of stomatal complexes that appears to determine the plasticity in this process, with premature expression preventing the manifestation of the stem cell divisions of Ms, and late expression allowing these cells to undergo amplifying divisions before the transition to GMC.

But how can *AtMUTE* inhibit the stem character of Ms? A recent study has shown that AtMUTE cooperates with epigenetic machinery to regulate chromatin remodeling and therefore, cell fate specification through transcriptional regulation (Liu *et al*., 2024). In addition, the regulation of the stem cell character of Ms depends on the heterodimerization of AtMUTE with the functionally redundant bHLH proteins ICE1 (also known as SCREAM) and SCREAM2 (Kanaoka *et al*., 2008). What is not known is whether this physical interaction between these bHLH proteins is necessary to regulate chromatin remodeling and/or gene transcription.

## *ZmMUTE* and *OsMUTE* define a pre‐GMC stage

In grasses, including maize and rice, the SPC that arises from the asymmetric division of a protodermal cell undergoes a single and symmetric cell division producing a cell that gives rise directly to the paired GCs (Stebbins & Shah, 1960; Serna, 2011; Hepworth *et al*., 2018; Wang *et al*., 2019; Wu *et al*., 2019; Nunes *et al*., 2020). Because Ms have been defined as stem cells that undergo several amplifying divisions before transition to GMC fate (Bergmann & Sack, 2007; Pillitteri & Dong, 2013; Han & Torii, 2016), does this mean that grasses lack Ms? If the answer is yes, considering that *AtMUTE* represses the stem cell character of Ms, mutations in their orthologs, *BZU2/ZmMUTE* and *OsMUTE*, should not trigger phenotypes like those of *atmute*. On the contrary, if the answer is negative, the repression of *MUTE* expression or activity in grasses may resume the self‐renewal capacity of their pre‐GMCs.

The loss‐of‐function *bzu2/zmmute* mutants have a higher leaf temperature than wild‐type (WT) plants, reflecting decreased transpiration (Wang *et al*., 2019). In addition, their photosynthetic activity is also decreased (Wang *et al*., 2019). These lethal physiological changes are a direct consequence of the defects in stomatal development. Indeed, the stomatal precursors of the *bzu2/zmmute* mutant, instead of symmetrically dividing to produce a pair of GCs, undergo excessive, randomly oriented and/or asymmetric divisions that give rise to short columns of two, three or even four elongated and undifferentiated cells (Fig. 1c; Wang *et al*., 2019). Unlike *AtMUTE* expression, *BZU2/ZmMUTE* is expressed in all young SPCs from the timeframe of SMC establishment (Wang *et al*., 2019). Therefore, *BZU2/ZmMUTE* expression is defining a stage which could be equivalent to the stage in which the last Ms of Arabidopsis are formed.

Like the *bzu2/zmmute* mutant, the lethal loss‐of‐function *c‐osmute* mutant exhibits also short columns of two cells, produced by misoriented and/or asymmetric cell divisions, instead of two dumbbell‐shaped GCs (Fig. 1c; Wu *et al*., 2019). Consistent with this phenotype, *OsSPCH1* and *OsSPCH2*, which control early steps of stomatal development (Liu *et al*., 2009; Wu *et al*., 2019), were expressed in *c‐osmute* (Wu *et al*., 2019), and *OsFAMA*, which is required for GC differentiation (Liu *et al*., 2009; Wu *et al*., 2019), was downregulated (Wu *et al*., 2019). Both transpiration and photosynthetic activity of the *c‐osmute* mutant, like those of the *bzu2/zmmute* mutant (Wang *et al*., 2019), must also be dramatically affected. In contrast to *AtMUTE* and like *BZU2/ZmMUTE*, *OsMUTE* expression was observed in all the stomatal precursors from the moment SMC divides (Wu *et al*., 2019). Therefore, *OsMUTE* expression, like *BZU2/ZmMUTE*, defines also a pre‐GMC stage.

The early expression of *BZU2/ZmMUTE* and *OsMUTE* strongly suggests that these genes prevent the manifestation of self‐renewal behavior of the pre‐GMCs, accelerating their immediate transition to the GMC fate. However, because both *bzu2/zmmute* and *c‐osmute* not only replace their stomata by short columns of cells but also lack SCs (Wang *et al*., 2019; Wu *et al*., 2019), the observed defects of the SPC division could simply be a consequence of the absence of SCs. Interestingly, although all stomata in *c‐osmute* are replaced by small cells that lack SCs (Wu *et al*., 2019), some complexes of *bzu2/zmmute* (*c*. 5%) developed one single SC (Wang *et al*., 2019). Considering that all stomata in *bzu2/zmmute*, including those that have one SC, were replaced by columns of undifferentiated cells (Wang *et al*., 2019), it seems that the defect of the SPC division is not a consequence of the absence of SCs, but of the absence of *BZU2/ZmMUTE* activity. Thus, the expression of *BZU2/ZmMUTE* and probably *OsMUTE* not only defines but also promotes a stage that may be equivalent to that in which the last M are formed in Arabidopsis.

## *BdMUTE* or *BdFAMA* are required to advance to a pre‐GMC stage

The *subsidiary cell identity defective* (*sid/bdmute*) mutant, with alterations in the Arabidopsis *MUTE* orthologue *BdMUTE*, in addition to having impaired other steps of stomatal development (Serna, 2025), undergoes misoriented divisions in *c*. 30% of the SPCs, aborting the formation of GCs (Fig. 1c; Raissig *et al*., 2017). However, in contrast to both *bzu2/zmmute* and *c‐osmute* mutants, which exhibit a complete lack of stomata (Wang *et al*., 2019; Wu *et al*., 2019), the remaining 70% of the stomatal precursors of the *sid* mutant produce dicot‐like stomata. Because of this, it is fully viable and fertile, although its gas exchange capacity and stomatal diffusion capacity are also affected (Raissig *et al*., 2017). The *BdMUTE* promoter is also induced early from the timeframe of SMC establishment (Raissig *et al*., 2017). Therefore, *BdMUTE* expression also defines a stage that could be equivalent to the stage in which the last Ms of Arabidopsis are formed.

Because the *sid/bdmute* mutant also lacks SCs (Raissig *et al*., 2017), the observed defects in SPCs could simply reflect a consequence of the absence of SCs. Interestingly, a line expressing *3×GFP‐BdMUTE* (the weakest expressing one) in the *sid/bdmute* background almost completely rescued paired GCs formation but failed to recruit SCs (Spiegelhalder *et al*., 2024). This indicates that the misoriented divisions of the stomatal precursors of the *sid/bdmute* mutant are not due to the absence of SCs, but to the *sid/bdmute* mutation. In agreement with this, although another of the lines expressing *3×GFP‐BdMUTE* (the highest expressing one) almost completely rescued the *sid/bdmute* phenotype, with the mutant exhibiting 98% of complexes identical to those of WT plants, a considerable number of GMCs, at the time of their division, lacked adjacent SCs because the recruitment of SCs was delayed (Spiegelhalder *et al*., 2024). Therefore, the lack of SCs does not trigger the defects of the SPC division in the *sid/bdmute* mutant, and such defects are a consequence of the absence of BdMUTE activity. It is then likely that premature expression of *BdMUTE*, like those of *ZmMUTE* and *OsMUTE*, represses cell divisions and perhaps the expression of a possible stem cell character of the pre‐GMC, accelerating its transition to GMC fate without the need to undergo amplifying divisions. Because grass *MUTE* genes function by correctly orienting the division plane of their stomatal precursors (Raissig *et al*., 2017; Wang *et al*., 2019; Wu *et al*., 2019; Spiegelhalder *et al*., 2024), it cannot be ruled out that the primary role of these genes is to change the orientation of cell division, from transverse to longitudinal, which in turn could regulate the cell division capacity.

Interestingly, a severe double *bdmute*; *bdfama* fails to produce any stomata and instead develops to short columns of undifferentiated cells like those of *bzu2/zmmute* or *c‐osmute* mutants (McKown *et al*., 2023). This is telling us that *BdFAMA*, in addition to positively regulating the transition from GMC to GC, also represses the stem cell character of the pre‐GMC. Consistently, *BdFAMA* expression is not restricted to the last stage of stomatal development, but its expression, starting during SC recruitment, overlaps with those of *BdMUTE* (Raissig *et al*., 2017; McKown *et al*., 2023). Assuming that grass *FAMA* function is conserved, the fully penetrant phenotype of both *bzu2/zmmute* and c*‐osmute* mutants suggests that in maize and rice, *FAMA* expression may depend on MUTE activity. In agreement with this, both *bzu2/zmmute* and *c‐osmute* barely express *FAMA* (Wang *et al*., 2019; Wu *et al*., 2019). Therefore, this MUTE‐dependent activation of *FAMA* could explain the complete abortion of stomatal development in the *bzu2/zmmute* and *c‐osmute* mutants. *BdMUTE* not only represses but also promotes stem cell divisions, which depend on the cell type. Indeed, overexpression of *BdMUTE* induces polarized SMC‐like divisions, giving rise to stomata with multiple layers of SCs (Raissig *et al*., 2017). This indicates that *BdMUTE*, in SMCs, promotes rather than ends stem cellness. The formation of similar cells throughout the epidermis when *OsMUTE* is overexpressed (Wu *et al*., 2019) could also point in the same direction.

## Concluding remarks and future perspectives

Ms possess a stem cell‐like character, reiterating asymmetric amplifying divisions. This has led us to propose that protodermal cells directly produce GMCs, and that grasses therefore lack Ms (Peterson *et al*., 2010; Hepworth *et al*., 2018; Serna, 2020). However, the phenotype of both *bzu2/zmmute* and *c‐osmute*, where all stomata are replaced by short columns of undifferentiated cells (Wang *et al*., 2019; Wu *et al*., 2019), suggests that maize and rice may have Ms, and that an early expression of both *BZU2/ZmMUTE* and *OsMUTE* may repress their stem cell divisions. Consistently, these genes are expressed in all young stomatal precursors from the stage of SMC establishment or division (Wang *et al*., 2019; Wu *et al*., 2019). Although the *sid/bdmute* mutant phenotype is not fully penetrant (Raissig *et al*., 2017), the same seems to occur in Brachypodium. The early expression of *BdMUTE* in all SPCs from the timeframe of SMC establishment (Raissig *et al*., 2017) suggests that it may repress the manifestation of their stem cell character. Therefore, the early expression of grass *MUTE* immediately depletes the potential stem cell pool. Delving into what determines this early expression would provide fundamental insights into the cellular biology of self‐renewal and the mechanisms that not only initiate but also regulate stem cell identity.

In Arabidopsis, the bHLH transcription factor SPEECHLESS (SPCH) promotes the transition of protodermal cells to MMC and their subsequent asymmetric divisions (MacAlister *et al*., 2007; Pillitteri *et al*., 2007; Lau *et al*., 2014). Grasses have two copies of SPCH (Raissig *et al*., 2016; Wu *et al*., 2019). The duplication of *SPCH* in this group of plants was not well understood, mainly because grasses were thought to lack Ms. Assuming that they have Ms, it is likely that grass *SPCHs*, redundantly and through strategies that differ from those of Arabidopsis *SPCH* (McKown & Bergmann, 2020), promote the transition of protodermal cells to MMC, and that the early expression of grass *MUTE* prevents grass *SPCHs* subsequent function. The function of the signaling peptides *EPIDERMAL PATTERNING FACTOR 1* (*EPF1*) and *EPF2* in Arabidopsis is also well known. While *EPF2* negatively regulates entry divisions and subsequent M activity (Hara *et al*., 2009; Hunt & Gray, 2009), *EPF1* predominantly regulates the M‐to‐GMC transition (Hara *et al*., 2007; Hunt & Gray, 2009). As it was assumed that grasses lacked Ms, the function of *EPF1/2*‐like peptides was thought to be also divergent between Arabidopsis and grasses (Chater *et al*., 2017). The questioning of the presence of Ms in grasses also questions such divergence. In addition, heterologous and homologous overexpression of *EPF1/2* from grasses negatively regulates stomatal development (Hughes *et al*., 2017; Lu *et al*., 2019; Jangra *et al*., 2021), suggesting that the function of these genes, at least in part, is conserved.

The stomatal complex has been fine‐tuned by several innovations, including incorporation of multiple asymmetric stem cell divisions in stomatal precursors to create a variety of stomatal distributions. For example, amplifying divisions, associated with the development of anisocytic stomatal complexes, are known to occur in some Piperales (Rudall, 2023). Therefore, it is likely that grasses lost their M function due to the early expression of grass *MUTE* genes, which possibly enhanced gas exchange. Furthermore, the function of *MUTE* genes appears to have changed over the course of evolution, as evidenced by the fact that the *Kalanchoe laxiflora MUTE* genes (*KlasMUTEs*) promote rather than repress Ms divisions (Cheng *et al*., 2024). Understanding the emergence and evolution of Ms, as well as the molecular basis of these changes in the function of *MUTE* genes, is an exciting challenge for the future.

## Competing interests

None declared.
